# Unique Raman Spectroscopic Fingerprints of B-Cell Non-Hodgkin Lymphoma: Implications for Diagnosis, Prognosis and New Therapies

**Published:** 2018-01-18

**Authors:** B Shiramizu, R Oda, N Kamada, MA Garcia, T Shieh, TA Maeda, SY Choi, E Lim, A Misra

**Affiliations:** 1 Department of Tropical Medicine, Medical Microbiology, and Pharmacology, John A. Burns School of Medicine, Hawaii, USA; 2 Department of Pediatrics, John A. Burns School of Medicine, Hawaii, USA; 3 Department of Molecular Biosciences and Bioengineering, John A. Burns School of Medicine, Hawaii, USA; 4 Hawaii Institute of Geophysics and Planetology, University of Hawaii, Hawaii, USA; 5 Biostatistics Core, Department of Complementary and Integrative Medicine, John A. Burns School of Medicine; University of Hawaii, Hawaii, USA

**Keywords:** Raman spectroscopy, Non-Hodgkin lymphoma, Lymphoma, B-cell lymphoma, Pediatric cancer

## Abstract

**Objective:**

Raman spectroscopy is a non-invasive laser-based technique that identifies molecular chemical composition of tissues and cells. The objective of the work was to demonstrate that unique Raman spectroscopic fingerprints of B-cell non-Hodgkin lymphoma cells could be distinguished from normal B-cells.

**Methods:**

Normal B-cells and B-cell non-Hodgkin lymphoma cells were mounted on aluminum slides and analyzed by Raman spectroscopy using Asymmetric Least Squares and Principal Component Analysis.

**Results:**

Clustering by Principal Component Analysis differentiated normal B-cells from B-cell non-Hodgkin lymphoma cells as well as between the different B-cell non-Hodgkin lymphoma cell types.

**Conclusions:**

Raman spectroscopy technology provided a different paradigm in analyzing tumor cells which could be used for diagnosis as well as contribute new information on unique characteristics of cancer cells to understand pathogenesis and potential novel treatments.

## Introduction

Often times, tools and criteria that are used to diagnose cancer from specimens lead to understanding pathogenic mechanisms which potentially provide insight into novel interventions. Recent advances in targeted therapy for childhood Non-Hodgkin lymphoma (NHL) stemmed from refinements in diagnosis and understanding NHL biology [1,2]. Although NHL treatment results in relatively good prognosis for children and adolescents, challenges continue to exist to further improve outcome [3,4]. Identifying novel tools to interrogate malignant cells could provide new paradigms for discovering innovative interventions. New technologies which provide biochemical analysis of specimens without fixatives, markers or stains could also pave the way to improve diagnostic paradigms.

Raman spectroscopy (RS) has been used to analyze malignant tissues and could be employed to advance NHL discovery and treatment [5-9]. RS is a laser-based technique that characterizes chemical molecular composition [10]. The laser effect on tissue results in inelastic scattering of photons by molecular bond vibrations and a portion of the incident photons is scattered resulting in a shift toward lower frequencies. The resulting energy difference between the incident and scattered photons corresponds to the vibrational energy of tissue-specific molecular bonds. The RS obtained from specimens results in intrinsic molecular fingerprints revealing information about DNA, protein and lipid content.

Raman spectroscopy relies on Raman scattering of radiation fractions by molecules from an incident beam based on chemical structures of molecules [11,12]. It has been used in cervical cancer studies not only as a diagnostic tool but in understanding disease at the molecular level [5,12-14]. It is possible to calculate the vibrational energy difference by the frequency of incident light and measuring frequency of Raman scattered light. This energy, the Raman shift, is expressed in wavenumbers (cm^−1^) in a plot known as the Raman spectrum. Ramos et al. recorded the Raman spectrum of a cervical cancer cell line which provided a unique fingerprint makeup of chemicals and molecules of the cancer cells [12].

As a non-destructive rapid diagnostic tool, RS has the potential to differentiate malignancies [5,6,10]. Small-round blue cell tumors in children include NHL, neuroblastoma, Ewing Sarcoma and rhabdomyosarcoma which typically require a combination of immunohistochemical staining, fluorescence in situ hybridization and karyotyping for definitive diagnosis [15]. Occasionally, NHL diagnosis involves further assessment to identify the subtype (B-cell, T-cell, large cell) of NHL [1,2,4]. RS detects chemical signatures of cells and tissue and could potentially be used to quickly and accurately diagnose NHL subtypes in real-time [6,8]. In this feasibility study, RS was used to compare unique RS fingerprints of B-cell non-Hodgkin lymphoma (B-NHL) cells from normal B-cells. This type of lymphoma is one of the most common in children thus will provide the initial background as the foundation for future studies if unique RS fingerprints are discovered [1].

## Methods

### Cells and specimen preparation

The study was approved in accordance with the University of Hawaii Institutional Review Board. B-NHL cell lines (Ramos and CA46) were obtained from American Type Culture Collection (ATCC, Manassa, VA) and cultured with RPMI 1640 medium and fetal calf serum. The cells were washed and re-suspended in 0.9% saline solution. Normal B-cells were isolated from peripheral blood using a negative selection Robosep kit (EasySep Human B-Cell Isolation Kit, Stemcell Technologies, Cambridge, MA) and re-suspended in saline solution as noted above.

### Raman spectroscopy of cells

RS was measured using a micro-Raman RXN system (KOSI, Inc., Ann Arbor, MI) utilizing a 785 nm laser and automated xyz microscope stage with cells mounted on aluminum reflective slides [7,9]. Polished aluminum sheets (Anomet. Inc., Ontario, Canada) of 0.5 mm thickness were cut and cleaned with methanol [16]. Cells were placed directly on the aluminum substrates for RS analysis. A 50 μm slit width was used for measuring the RS of cells. Each RS was collected for 60 seconds with 10mW laser power. Raman images were created by measuring RS at 100 points on a 120 μm × 80 μm grid at each point recorded twice with 30 mW of laser power and 10 second integration time.

### Data analysis

Asymmetric Least Squares (AsLS) and Principal Component Analysis (PCA) were applied to the spectral data (n=20 spectra per cell type). RS were collected within the Running title: Raman spectroscopy of B-cell lymphoma Page 6 spectral region from 600 to 1800 cm^−1^, which represented the molecular fingerprint region that provides information on biological constituents of cells [10,17]. Spectra were background corrected and individual spectra were then normalized with respect to the total area under the Raman curve. The spectra from each cell type were averaged to obtain a mean RS of the cell type. Subsequently, PCA was performed to extract inherent structures of the spectra from cells and to compare different B-NHL cells and normal B-cells against each other. A scree plot and eigenvalue of ≥ 1 were used to identify the number of PC. Additionally, k-nearest neighboring (KNN) method was applied to confirm the discrimination capability results from PCA [18]. A continuous baseline correction was performed on each Raman spectrum using AsLS, thereafter spectra were smoothed [6,11,19]. Raman spectra were collected from several cells of samples and representative of the response from each cell line subtypes, were used to identify the most significant bands.

PCA analysis was performed on spectra after the baseline correction, smoothing and area normalization procedures. The result of a PCA analysis performed on a given data set is a vector which contains the relevance of principal components classified as a function of their variance. Usually, most of the variance is contained in the first three principal components PC1, PC2 and PC3. Thereafter, analytical algorithm is applied to the PCA results in order to separate the data into statistically similar groups. Statistical analysis was performed using custom scripts written in Matlab©.

## Results

**Figure 1:**
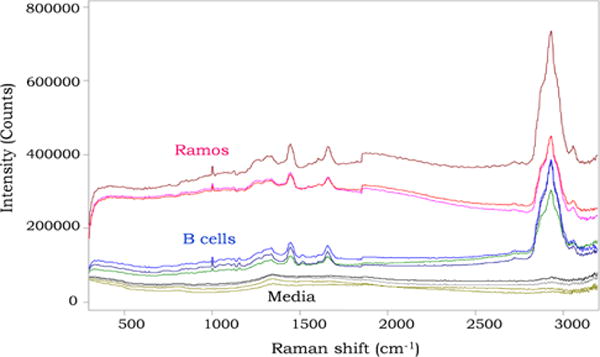
Representative Raman spectra (RS) fingerprints of cells and media. RS fingerprints of Ramos cells, normal B-cells and cell culture media.

RS fingerprints were obtained from cell lines and normal cells. When compared and processed, the RS of cells and media identified distinct peaks however differences between cell types were difficult to visualize (Figure 1). The RS peaks represented fingerprints ranging from DNA/protein concentrations and saccharide bonds [10]. However to better delineate unique RS fingerprint differences between cell types, additional analyses were performed.

### Asymmetric least squares (AsLS) and principal component analysis (PCA)

**Figure 2:**
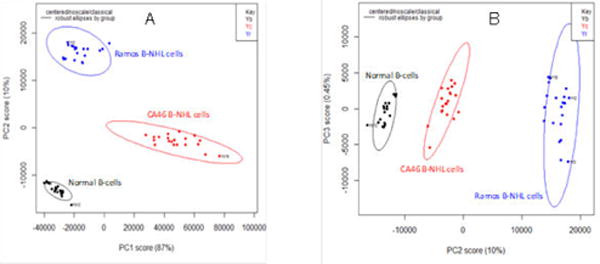
Principal component analyses (PCA) score plots of normal B-cells versus B-cell non-Hodgkin lymphoma (B-NHL) cells. A) Principal component (PC)1 *vs.* PC2 (PCA1) comparing normal B-cells, Ramos B-NHL cells (blue) and CA46 B-NHL cells (red), PC1 and PC2 make up 97.0% variability; B) PC2 *vs.* PC3 (PCA2) comparing normal B-cells (black), Ramos B-NHL cells (blue) and CA46 B-cells (red), PC2 and PC3 make up 10.45% variability.

The RS from normal B-cells and B-NHL cells were baselined using AsLS followed by PCA which the scree plot identified 3 PCs to account for 97.45% of variability in the data [20]. The first PC explained the greatest amount of variance (87.0%) while the second PC explained 10.0% of variance (Figure 2A and 2B) identified the distinct cells as defined by the scree plot showing clear clusters amongst the distinct cells. KNN was then used to confirm the discrimination capability results from the three PCs [18]. KNN showed an accuracy and specificity of 100%.

## Discussion

RS is a technique which has been used to identify malignant tissue [5,7]. However, applying RS technology to tissue requires probing relatively large specimen volumes to average the information from large numbers of cells [21,22]. RS fingerprints contain spectral bands representing molecular modes of vibration of molecules within the tissue. Identifying unique RS fingerprints of malignant cells and normal cells will be important to further understand pathogenesis and mechanisms of malignant transformation in order to discover novel treatment strategies. The current study provides data which could supplement cancer diagnosis by using unique RS fingerprints to not only distinguish cancer cells but possibly learn more about the pathogenic characteristic of the malignant cell [5,6,13,14]. Integrating RS diagnostic fingerprinting in routine cancer diagnostic paradigms could be an innovative approach which has the potential to enhance the translation of RS towards new diagnostic prospects [14].

The RS fingerprints of normal B-cells and B-NHL cells had similar phenotypes which were consistent with previous RS from human lymphocytes [6,23]. The spectra of the cells shared similar peaks which could be assigned to cellular constituents (DNA/RNA, proteins, lipids, carbohydrates) [6].

The data focused on the PCA between normal B-cells and B-NHL cells which was used to reduce the large amount of RS information into principal components. The scattered plots which were generated showed clusters of points distinguishing the two B-NHL cell lines from normal B-cells. While the PCA score plots showed clear distinctions between the PCs, PCA1 (PC1 *vs.* PC2) showed the maximal separation between all three cell types. The use of PCA in the analytical algorithm had the potential to provide a more clinical translational approach to deciphering the raw data [6,11,19].

The study demonstrated that RS is capable of identifying and distinguishing malignant B-NHL cells from normal B-cells. A limitation of the study is that the population of cells Running title: Raman spectroscopy of B-Cell lymphoma Page 9 which were analyzed were pure populations. Therefore the raw RS data and PCA data represented RS fingerprints of pure cells. Future plans will be to assess RS fingerprints of; mixed populations of cells. The focus of the current study was to assess the feasibility of identifying unique RS fingerprints of pure populations of malignant cells compared to normal cells. Another limitation of the study is that the cancer cells which were analyzed were focused on childhood B-cell lymphoma which is one of the most common childhood lymphomas diagnosed in pediatrics. The study was initially carried out to assess the feasibility of identifying RS fingerprints in this relatively common childhood lymphoma. Future studies will expand the subtypes of other childhood lymphomas including T-cell and large B-cell lymphomas. With the aid of statistical models, discriminating different malignant cells from normal B-cells was possible based on the specific biochemical information which was delineated from the RS data. The PCA discrimination of the different B-cell lymphoma cells from normal cells was limited because of the 100% purity of the cell populations. The variance was assumed because of the cell populations. Further discrimination in mixed cell cultures and analyses will better discern the applicability of RS fingerprinting in future planned experiments.

Results of the study show promise that RS fingerprinting of B-NHL could be feasible. While this initial study was designed to initially assess one of the most common lymphoma subtypes in children, other lymphoma cell types will need to be analyzed to determine differences in Raman spectra fingerprints. This has implications for future diagnostic use and prognosis as well as identifying new therapeutic targets for B-NHL.
